# Changes in Retinal Function and Cellular Remodeling Following Experimental Retinal Detachment in a Rabbit Model

**DOI:** 10.1155/2017/4046597

**Published:** 2017-04-09

**Authors:** Tilda Barliya, Ron Ofri, Shai Sandalon, Dov Weinberger, Tami Livnat

**Affiliations:** ^1^Division of Ophthalmology, Rabin Medical Center, Beilinson Campus, Petah Tikva, Israel; ^2^Laboratory of Eye Research, Felsenstein Medical Research Center (FMRC), Rabin Medical Center, Petah Tikva, Israel; ^3^Koret School of Veterinary Medicine, The R. H. Smith Faculty of Agriculture, Food and Environment, The Hebrew University of Jerusalem, Rehovot, Israel; ^4^Sackler School of Medicine, Tel Aviv University, Tel Aviv, Israel; ^5^The Israeli National Hemophilia Center, Sheba Medical Center, Tel Hashomer, Israel

## Abstract

*Purpose.* To explore functional electroretinographic (ERG) changes and associated cellular remodeling following experimental retinal detachment in a rabbit model. *Methods.* Retinal detachment was created in ten rabbits by injecting 0.1 ml balanced salt solution under the retina. Fundus imaging was performed 0, 3, 7, 14, and 21 days postoperatively. ERGs were recorded pre- and 7 and 21 days postoperatively. Eyes were harvested on day 21 and evaluated immunohistochemically (IHC) for remodeling of second- and third-order neurons. *Results.* Retinal reattachment occurred within two weeks following surgery. No attenuation was observed in the photopic or scotopic a- and b-waves. A secondary wavefront on the descending slope of the scotopic b-wave was the only ERG result that was attenuated in detached retinas. IHC demonstrated anatomical changes in both ON and OFF bipolar cells. Bassoon staining was observed in the remodeled dendrites. Amacrine and horizontal cells did not alter, but Muller cells were clearly reactive with marked extension. *Conclusion.* Retinal detachment and reattachment were associated with functional and anatomical changes. Exploring the significance of the secondary scotopic wavefront and its association with the remodeling of 2nd- and 3rd-order neurons will shade more light on functional changes and recovery of the retina.

## 1. Introduction

Rhegmatogenous retinal detachment (RRD) is a serious condition associated with acute visual loss caused by anatomic displacement of the photoreceptor layer. RRD is often associated with permanent loss of vision which may be due to retinal remodeling even if reattachment occurred [1, 2]. The resulting degeneration of the photoreceptor layer is an important event that initiates downstream cellular changes throughout the retina. These morphological changes are often associated with impairment of retinal function [3] as can be seen by the loss of electroretinographic (ERG) responses, which often are proportional to the area of the detachment [4].

It was recently reported that the retina, like the central nervous system, has a significant capacity for remodeling its cellular architecture [5]. This remodeling process includes a wide range of changes, including the retraction of axons by rod photoreceptors, outgrowth of horizontal cell neurites, and Müller cell proliferation and structural reorganization [6]. In human RRD patients, it was found that upon successful reattachment, the photoreceptor outer segments almost fully regenerate and the retinal-pigmented epithelium (RPE) can achieve good contact with the retina [7]. This reattachment results in recovery of ERG function. However, if the outer nuclear layer (ONL), the outer plexiform layer (OPL), or the inner nuclear layer (INL) does not fully regenerate, the ERG deficits will persist [7]. It is therefore believed that a strong interplay exists between retinal remodeling and functional recovery following detachment and reattachment [6, 8].

In this study, we investigated the functional ERG changes and associated cellular remodeling following experimental retinal detachment and reattachment in a rabbit model. We report for the first time on the attenuation of a secondary wavefront on the descending slope of the scotopic b-wave, which was filtered to rule out oscillatory potential components. Persistent ERG changes and enhanced remodeling of the second- and third-order retinal neurons were observed following retinal detachment and reattachment.

## 2. Materials and Methods

### 2.1. Animal Model

This study was carried out in strict accordance with the recommendations in the Guide for the Care and Use of Laboratory Animals of the National Institutes of Health. The protocol was approved by the Committee on the Ethics of Animal Experiments of the Rabin Medical Center, Beilinson Campus, Israel (Protocol number 022-b5312_9/18/12).

Ten New Zealand albino male rabbits (Harlan Biotech Israel Ltd., Jerusalem, Israel) weighing 1.5–1.7 kg were used in this study. Animals were handled according to the recommendations of the ARVO statement for the Use of Animals in Ophthalmic and Visual Research and the Institutional Animal Care and Use Committee.

### 2.2. Retinal Detachment Procedure

All procedures were carried out in the right eyes while the left eyes served as controls. Animals were anesthetized by an intramuscular (IM) injection of ketamine hydrochloride (40 mg/kg) and xylazine (10 mg/kg) (Vetmarket, Shoham, Israel). The pupils were dilated with topical 0.5% phenylephrine hydrochloride, 0.5% tropicamide (Fischer Pharmaceutical Laboratories, Tel Aviv, Israel), and 1% atropine. Conjunctival peritomy and sclerotomy of 180° (from 0800 to 1400 hours) were performed at the corneoscleral limbus, followed by a two-port vitrectomy. An infusion port was made 1 mm posterior to the sclerocorneal limbus in the inferotemporal quadrant using a small gauge stiletto. A 25-gauge infusion cannula (Accurus Surgical System 25-gauge; Alcon, TX, USA) that delivered balanced salt solution (BSS; Alcon, Japan) was then inserted into the trocar cannula. The second port, created using the same methodology, was used for inserting a vitreous cutter into the superotemporal quadrant. Subsequently, we performed core vitrectomy, and retinal detachment was induced by a subretinal injection of 0.1 ml BSS using a 25G-soft-tip needle (Alcon, TX, USA). All operated eyes were treated with 5% chloramphenciol ointment at the end of the procedure. Clinical examination of the fundus was performed using an indirect ophthalmoscope (Vantage Plus Digital Indirect Ophthalmoscope©, Keeler Ltd., Windsor, UK) 0, 3, 7, 14, and 21 days postoperatively. The operated eyes were examined for the presence of retinal detachment, hemorrhage, and tears as well as lens opacity and other potential complications. Detachment area was quantified at different days postoperatively using ImageJ software (Fiji Imaging processing software http://fiji.sc/Fiji). Statistical analysis was conducted using *t*-test (SPSS software IBM, NY, USA).

### 2.3. ERG

ERGs were recorded a week before and 7 and 20 days after surgery. Following an overnight dark adaptation, the rabbits were anesthetized with an IM injection of ketamine (60 mg/kg) and xylazine (8 mg/kg) and the pupils were dilated with 0.5% tropicamide (Mydramide, Fischer Pharmaceutical Laboratories, Tel Aviv, Israel) at least 8 minutes before the beginning of the ERG session. Subcutaneous needles at the base of the left ear and at the lateral canthus of the recorded eye served as ground and reference electrodes, respectively. After application of topical anesthesia (oxybuprocaine 0.4%) and conductance medium (hydroxymethyl cellulose 1.4%), a Jet corneal active electrode was placed. Impedance was kept below 2k*Ω*. The operated eye was recorded first. Recordings were conducted using an integrated system (HMsERG, Ocuscience, NV, USA) with band-pass filter of 0.3–300 Hz. Flash stimuli and background adapting light were delivered unilaterally with a “miniganzfeld” dome placed about 3 cm from the recorded eye. The rabbit was positioned in lateral recumbency on a pillow, thus preventing light exposure of the unrecorded eye. Responses to the standard ISCEV protocol (Table 1) were recorded.

The raw traces were further low-pass filtered below 150 Hz and at 50 Hz. In the dark-adapted steps (1–3), the a- and b-wave amplitudes were measured from baseline to the first trough and from that trough to the next positive peak, respectively. Implicit times were measured from flash stimulus onset to the relevant peak or trough.

**Figure 1:**
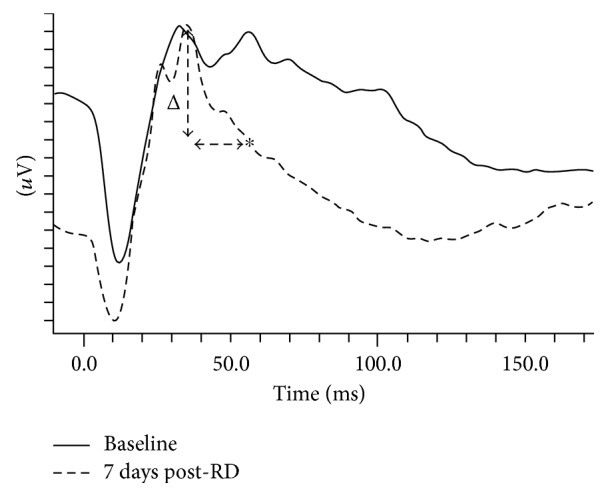
WDS measurement. Representative ERG traces (dark adapted, 10 cd∗s/m^2^) of the same eye before (solid line) and 7 days postretinal detachment (dashed line). The traces are aligned at the b-wave peaks for ease of comparison; therefore the baseline (i.e., zero *μ*V) value of the vertical axis is not designated. One vertical increment = 10 *μ*V. The WDS difference (vertical dashed arrow) is defined as the amplitude difference between the b-wave peak and the WDS peak. In many operated eyes, in which the WDS was abolished, the WDS was set as the recorded potential measured at the implicit time of the baseline WDS (designated with asterisk). Δ = difference; WDS = wavefront of the descending slope; RD = retinal detachment.

A wavefront riding on the descending slope of the b-wave (WDS) was observed in two out of the three scotopic recordings. To analyze this peak, the amplitude difference between the b-wave peak and the WDS was measured and analyzed (Figure 1). In the light-adapted steps, only the b-wave was analyzed due to noise level a-waves.

### 2.4. ERG Statistical Analysis

Since the same individual rabbits were repeatedly recorded throughout the study, ANOVA with repeated measures was used. When ANOVA revealed significant interaction between variables, a *t*-test with the *p* value corrected to the Bonferroni criterion (i.e., 0.05 divided by the number of repetitions) was performed to identify the outstanding variable. Error bars and “±” indicate SD.

### 2.5. Immunohistochemistry (IHC)

At the end of the 3-week follow-up, animals were euthanized with an overdose of intracardiac pentobarbital injection (Vetmarket, Shoham, Israel). Both eyes were enucleated, fixed in Davidson solution for 24 hrs, dehydrated by increasing sucrose gradient, and cryopreserved in OCT. Serial 10 *μ*m cryosections were evaluated for the expression of PKC*α* (rod bipolar cells), iGluR4 (OFF bipolar cells), mGluR6 (ON bipolar cells), GFAP (activated Müller cells), calretinin (all amacrine cells), calbindin (horizontal cells), and Bassoon (synaptic ribbon). A multistep-staining protocol was designed based on the characteristics of the primary antibodies used. Briefly, slides were permeabilized with 0.5% TritonX-100 (TBS) for 10 minutes, followed by blocking with either goat or donkey antiserum in BSS for 1 hr, washed several times, and incubated with the primary antibody for 1 hr. The primary antibodies used in this study were mouse monoclonal PKC*α* MC5 (ThermoScientific®, Rockford, IL, USA, 1 : 100), goat polyclonal iGluR4 (Abcam, Cambridge, MA, USA, 1 : 100), guinea pig polyclonal mGluR6 (Abcam, Cambridge, MA, USA, 1 : 100), chicken polyclonal GFAP (Abcam, Cambridge, MA, USA, 1 : 100), rabbit polyclonal calretinin (Abcam, Cambridge, MA, USA, 1 : 100), and mouse monoclonal calbindin (Abcam, Cambridge, MA, USA, 1 : 100). Tissue sections were then rinsed several times and incubated for 1 hr with donkey antimouse Alexa Fluor 488, donkey antigoat Alexa Fluor 568, goat antiguinea pig 647, goat antichicken 568, antirabbit Alex Fluor 488, or goat antimouse 568, respectively (Invitrogen, Grand Island, NY, USA). Slides were washed with BSS several times and incubated with a secondary antibody against the respective primary antibody. A similar procedure was followed for all antibodies. The following day, cell nuclei were counterstained with DAPI (Invitrogen, Grand Island, NY, USA) and mounted with mounting medium and images were captured digitally using either the Axio Imager Apotome microscope (Zeiss, Göttingen, Germany) or the Confocal Laser Scanning microscope (Leica TCS SP8, Buffalo Grove, IL, USA).

## 3. Results

### 3.1. Fundus Examination

**Figure 2:**
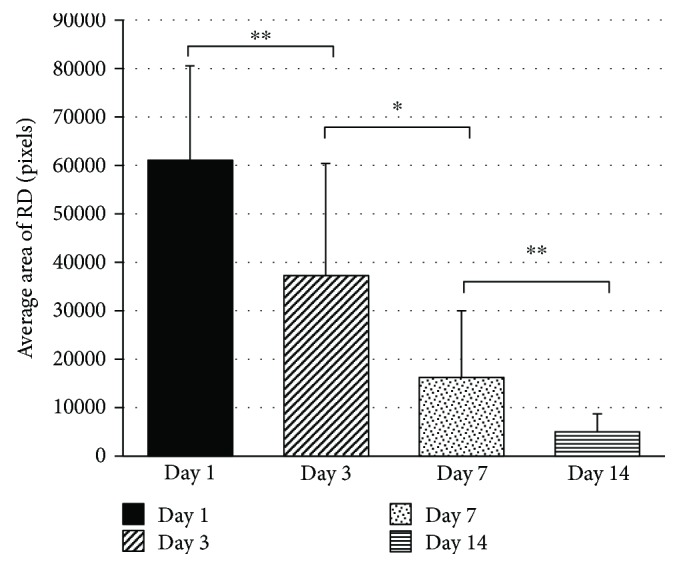
Illustration of the area of the detachment at different time course. ^∗^Statistically significant, *p* < 0.01. ^∗∗^Highly statistically significant, *p* < 0.005.

Funduscopic examination was performed to confirm the formation of a peripheral detachment (30% detachment) with a visible tear in all operated eyes on the day of the surgery. Other than one eye which had a focal opacity in the lens postsurgery and prevented thorough examination of the eye, no other evidence of intraocular or lens opacity was observed throughout the 3-week follow-up period. Negligible hemorrhage was seen at the site of detachment in one eye immediately after surgery and 7 days postoperatively but had resorbed by the end of the experiment. A retinal detachment and its associated tear were clearly visible in the 8/10 operated eyes on day 7 postsurgery and in 6/10 of the eyes on day 14, though they were much smaller in size and somewhat difficult to detect. The tears were barely detectable at this time point. By the end of the third week, the detachment and tear were not visible in any of the operated eyes and the retinas seemed to be reattached. A small scar was seen at the site where the needle tip pierced the retina in the 3/10 eyes.  Figure 2 illustrates the area of the detachment at different time course. The area of RD created on day 1 of the surgery was highly consistent between the different animals in the study (30% detachment). A consistent and significant reduction in the area of RD (40% reduction) was seen on day 3 postsurgery (*p* < 0.005), 60% reduction on day 7 (*p* < 0.01), and another 70% reduction on day 14 (*p* < 0.05). Three weeks postsurgery, the retina seemed to be reattached and ergo no measurements could be applied. While retinal tear was visible on day 1 and thereafter as described in the manuscript, its size was not consistent (data was not shown). In addition, since the fundus images are 2D, we could not measure the height of the detachment.

**Figure 3:**
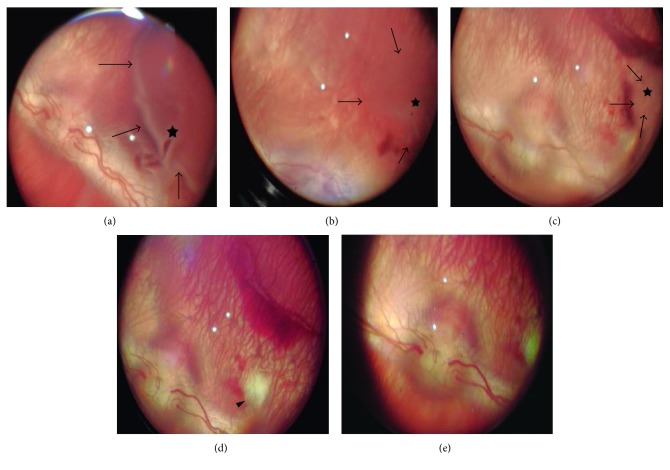
Representative fundus follow-up of retinal detachment in a single experimental eye. (a) 0, (b) 3, (c) 7, (d) 14, and (e) 21 days postdetachment. Retinal detachment (arrows), tear (asterisk), and local scar (arrowhead) are visible.

Figure 3 is a representative series of fundus images during the 3-week follow-up of an experimental eye.  Figure 3(a) shows a well-defined detachment inferior to the optic disc (arrows) with a small tear (asterisk) immediately after surgery. A major reduction in the size of the detachment was seen on day 3 (Figure 3(b)) and day 7 (Figure 3(c)), though both the detachment (arrows) and the tear (asterisk) were still clearly visible (resp.). A small scar was visible adjacent to the detached retina. On day 14 (Figure 3(d)), a further reduction in the size of the detachment occurred, making it barely visible (arrowhead), while the tear could not be seen. Three weeks postoperatively (Figure 3(e)), neither the detachment nor the retinal tear could be seen.

### 3.2. ERG

**Figure 4:**
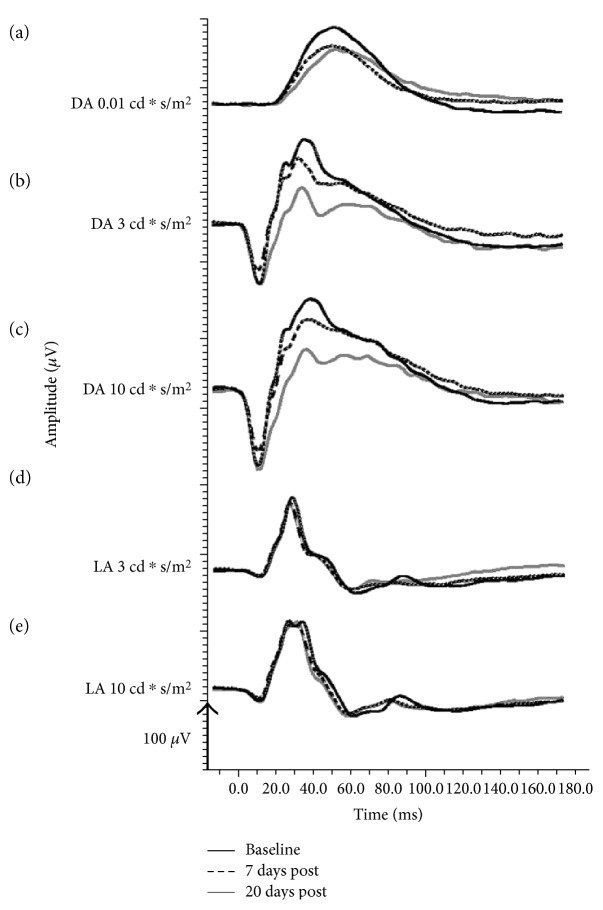
Representative traces in response to the five transient flash stimulations, recorded from a single eye before 7 and 20 days postretinal detachment. Note the WDS attenuation in panels (b) and (c). DA = dark adapted, LA = light adapted, and RD = retinal detachment.

Representative ERG traces of the operated eye of one rabbit before surgery and 7 and 20 days postoperatively are shown in Figure 4. The WDS was found in the two higher flash intensities of the scotopic recordings (Figures 4(b) and 4(c)). Note the postoperative nonsignificant decrease in the dark-adapted a-wave together with the attenuation of the WDS.

**Figure 5:**
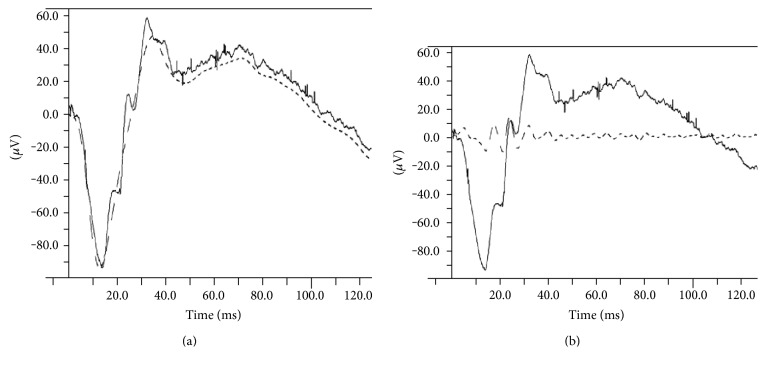
The WDS is not part of the oscillatory potential complex. (a) Low-pass filtering (<100 Hz) of a representative trace (3 cd∗ s/m^2^). The WDS is seen in both the unfiltered (solid line) and the filtered (dashed line) traces, while the oscillatory potentials are seen superimposed on the ascending limb of the unfiltered b-wave only. (b) High-pass filtering (>100 Hz, dashed line) of the same trace shown in panel (a), demonstrating no high-frequency components of the WDS, while the oscillatory potentials are clearly seen. The unfiltered trace is once again shown as a solid line.

A consistent wavefront superimposed on the descending slope of the b-wave was found in response to 3 and 10 cd∗s/m^2^ stimulation of the dark-adapted, intact retina (i.e., in the control eyes and baseline recordings of the experimental eyes). This wavefront, which we termed WDS, was not an oscillatory potential, as demonstrated by the analysis in Figure 5, which shows a raw (dark trace) and filtered (dashed trace) 3 cd∗s/m^2^ response. When the response was low-pass filtered under 100 Hz, the oscillatory potentials on the ascending slope of the b-wave are effectively smoothed, but the WDS remains unchanged (Figure 5(a)). However, when only 100 to 300 Hz signals are passed, the oscillatory potentials are apparent, yet no component of the WDS is isolated (Figure 5(b)).

**Figure 6:**
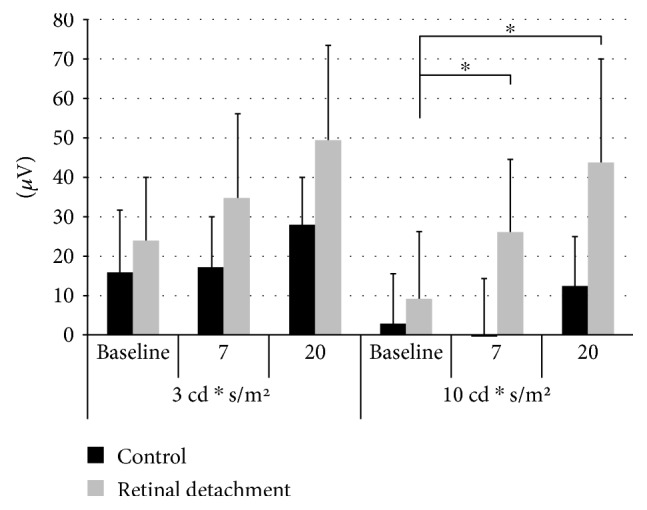
Mean ± SD amplitude differences between the b-wave and WDS peaks at three timepoints: preoperatively and 7 and 20 days postretinal detachment (*n* = 10). Considering all timepoints and flash intensities, the difference between the experimental and control eyes was significant (ANOVA with repeated measures, *p* < 0.05). Specifically, a significant increase in the WDS difference of RD eyes in response to the higher flash intensity was found, meeting the Bonferroni criterion (*p* < 0.016) at both 7 and 20 days postprocedure (∗). RD = retinal detachment.

The average amplitude difference between the b-wave and the WDS peaks is summarized in Figure 6. A higher flash intensity resulted in a decrease of this b-wave-WDS difference (i.e., more prominent WDS), regardless of the study group (ANOVA with repeated measures, *p* < 0.05). Compared to the control eyes, the b-wave-WDS difference of the operated eyes was higher at both postoperative recordings (7 and 20 days) and at both flash intensities (*p* < 0.05). This finding was more prominent in the higher flash intensity (10 cd∗s/m^2^), meeting the Bonferroni criterion, that is, *p* < 0.016 (Figure 6, right side). The implicit times of the WDS in the control eyes were 64.0 ± 6.3 and 62.8 ± 7.6 milliseconds for 3 and 10 cd∗s/m^2^, respectively. This parameter was not analyzed in the operated eyes due to complete attenuation of the WDS in many cases. There were no significant differences between the operated and control eyes in any other parameter (a- and b-wave amplitudes) of the scotopic and photopic ERG (*p* > 0.05, data not shown).

### 3.3. Remodeling of Bipolar Cells

**Figure 7:**
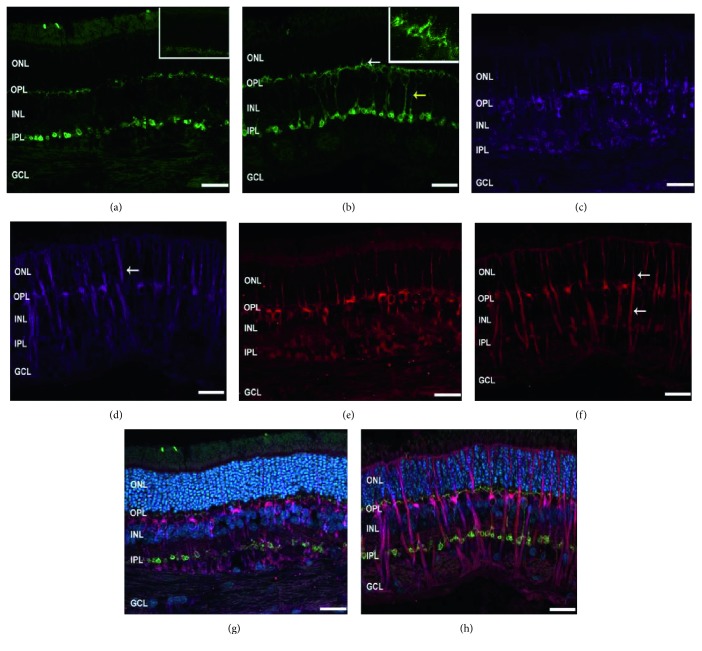
Remodeling of bipolar cells in a control (left column) and in a detached retina (right column). Representative images of retinal sections labelled with PKC for mixed bipolar cells ((a), (b), green), mGluR6 for on bipolar cells ((c), (d), purple), and iGluR4 for off bipolar cells ((e), (f), red). Merged images are illustrated, counter stained with DAPI ((g), (h), DAPI-blue). IS = inner segment, OS = outer segment, ONL = outer nuclear layer, INL = inner nuclear layer, OPL = outer plexiform layer, INL = inner nuclear layer, IPL = inner plexiform layer, and GCL = ganglion cell layer. Original magnification ×20, enlarged images of (a) and (b) at ×63 magnification.

To explore cellular retinal changes, we evaluated the expression pattern of markers for rod, ON and OFF bipolar cells.  Figure 7 is a representative triple-staining image of PKC*α* (rod bipolar cells), mGluR6 (ON bipolar cells), and iGluR4 (OFF bipolar cells). In the control retina (Figure 7(a)), PKC*α* labelling was present in the cell bodies of rod bipolar cells in the INL and their dendrites in the OPL (enlarged image, top right), with some faint staining of their axons in the IPL. In the experimental retina, however, dendrites of remodeled rod bipolar cells extend beyond their normal boundary into the ONL, which is normally free of these processes (Figure 7(b), white arrow). These cells also featured stronger staining of their cell bodies and axons (Figure 7(b), yellow arrow). The expression pattern of mGluR6 was mainly observed in the OPL of the control retina with some weak staining in the INL (Figure 7(c)). In the experimental retina, mGluR6 labelling was localized in the cell bodies and throughout the dendritic tips of the ON bipolar cells (Figure 7(d), white arrow). Interestingly, the labelling of iGluR4 was markedly enhanced in the neurite extensions in the ONL as well as in the axons spanning the INL and IPL (Figure 7(f), white arrows) of the experimental retinas compared to the control retinas (Figure 7(e)).

### 3.4. Expression Pattern of Bassoon, a Presynaptic Active Zone Protein

**Figure 8:**
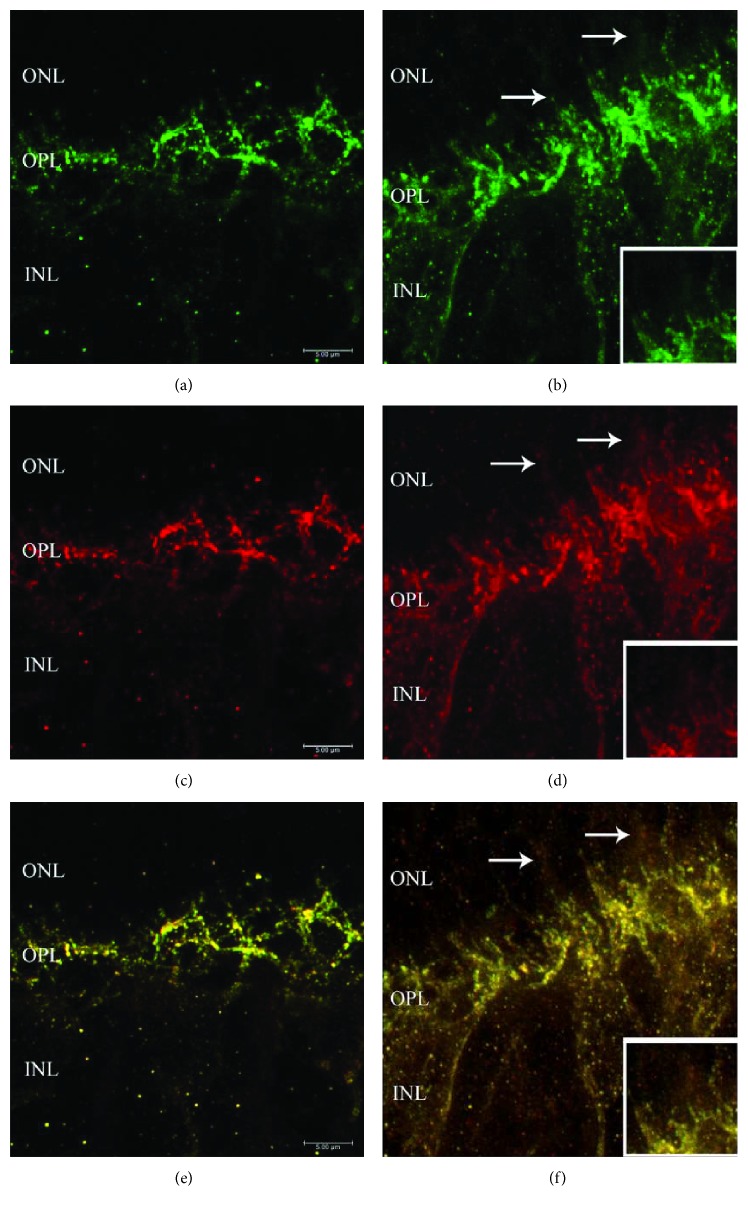
Expression of photoreceptor ribbon synapse protein Bassoon in a control (left column) and in an experimental retina (right column). Representative images of retinal sections labelled with PKC for mixed bipolar cells ((a), (b), green) and Bassoon for active ribbon synapse ((c), (d), red). Merged images are illustrated ((e) and (f)). ONL = outer nuclear layer, OPL = outer plexiform layer, and IPL = inner plexiform layer. Original magnification ×63. Higher magnification in the box (×100).

To further examine any changes associated with the contact and activity of bipolar cells, we looked at the paired PKC*α*/Bassoon profile which accounts for ON-rod bipolar cells and the photoreceptor synaptic ribbon, respectively. The results are summarized in Figure 8. In the control retina, the outer retina appeared to have a normal morphology, with bipolar cell dendrites (Figure 8(a), PKC-green) terminating near the OPL with coexpression of Bassoon-immunoreactive puncta which define the photoreceptor synaptic ribbon (Figure 8(c), Bassoon-red). In the experimental retina, however, dendrites were sprouting into the ONL and their axons extended into the INL (Figure 8(b), PKC-green, arrows, and higher magnification in the lower box). Moreover, we found many Bassoon-immunoreactive spots at the extended dendrites in ONL layer and not just localized at the end of dendrites which is indicative of the elongation and activation of the rod spherules (Figure 8(d) arrows, higher magnification in the lower box). Bassoon-immunoreactive spots were also observed in the axon and cell bodies in the IPL (data not shown). Merged images of colocalization are illustrated (Figures 8(e) and 8(f), resp.).

### 3.5. Amacrine and Horizontal Cell Remodeling

**Figure 9:**
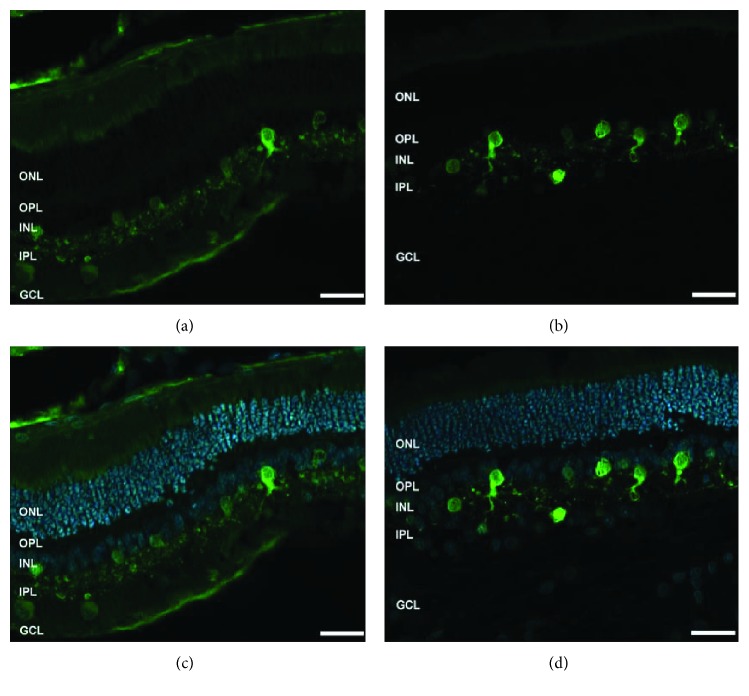
Expression pattern of all amacrine cells in the control and experimental retinas. Representative images of the control retinal sections ((a) and (c)) and detached retinas ((b) and (d)) labelled for calretinin (green) and counter stained with DAPI (blue). IS = inner segment, OS = outer segment, ONL = outer nuclear layer, INL = inner nuclear layer, OPL = outer plexiform layer, INL = inner nuclear layer, IPL = inner plexiform layer, and GCL = ganglion cell layer. Original magnification ×40.

Structural remodeling was observed in amacrine cells of patients with retinitis pigmentosa [9] and to some extent after retinal detachment-reattachment in a feline model [5]. We therefore sought to evaluate whether such remodeling also occurs in our rabbit model of retinal detachment.  Figure 9 is a representative image that shows the expression pattern of calretinin, an all-amacrine cell marker, in the control and experimental retinas. No major differences were observed in the calretinin-staining pattern between the eyes. In the control retinas, amacrine cell bodies were localized to the inner part of the INL and their stratification in the IPL was clearly demonstrated by the strong calretinin staining (Figures 9(a) and 9(c)). A similar staining pattern was observed in the experimental retinas (Figures 9(b) and 9(d)).

**Figure 10:**
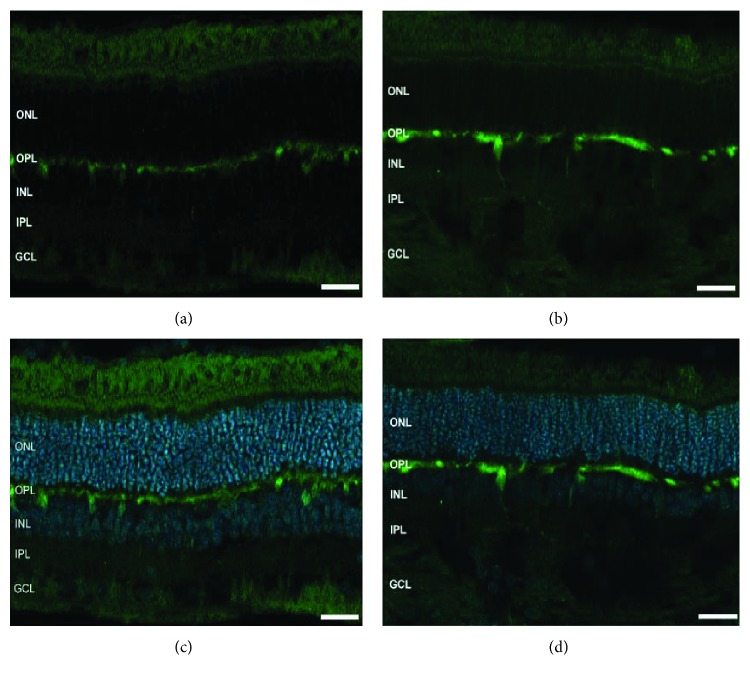
Expression pattern of horizontal cells in the control and experimental retinas. Representative images of the control retinal sections ((a) and (c) and detached retinas ((b) and (d)) labelled for calbindin (green) and counter stained with DAPI (blue). IS = inner segment, OS = outer segment, ONL = outer nuclear layer, INL = inner nuclear layer, OPL = outer plexiform layer, INL = inner nuclear layer, IPL = inner plexiform layer, and GCL = ganglion cell layer. Original magnification ×40.

Significant remodeling of horizontal cells was previously reported in both feline and rabbit models of retinal detachment [10, 11]. The expression pattern of calbindin, a marker for horizontal cells, was therefore evaluated (Figure 10). In the control retinas, strong labelling of horizontal cell stratification was observed in the OPL, with their cell bodies lying in the outer part of the INL (Figures 10(a) and 10(c)). A similar pattern was observed in the experimental eyes, where the ONL was devoid of any horizontal cell outgrowth (Figures 10(b) and 10(d)).

### 3.6. Müller Cell Activation

**Figure 11:**
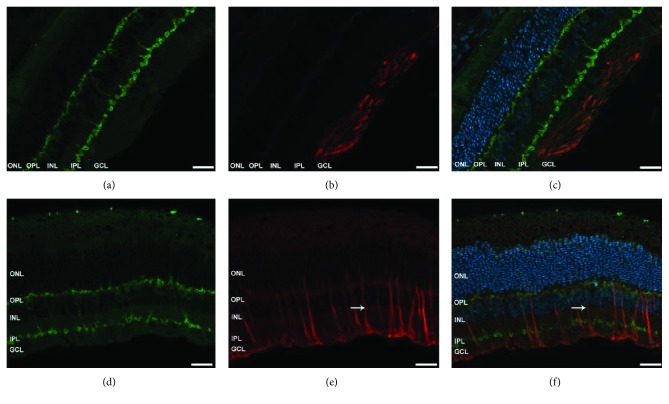
Expression pattern of GFAP in the control and experimental retinas. Representative images of the control retinal sections ((a), (b), and (c)) and detached retinas ((d), (e), and (f)) labelled for PKC*α* (green), GFAP (red) counter stained with DAPI (blue). IS = inner segment, OS = outer segment, ONL = outer nuclear layer, INL = inner nuclear layer, OPL = outer plexiform layer, INL = inner nuclear layer, IPL = inner plexiform layer, and GCL = ganglion cell layer. Original magnification ×40.

Retinal GFAP expression is mostly observed in degenerating injured and/or detached retinas [12]. In the control retinas, GFAP labelling was restricted to the ganglion cell layer (GCL), most likely to Müller cell end feet (Figures 11(b) and 11(c)). PKC*α* expression in the control retinas was found in bipolar cell bodies (Figure 11(a)). Double labelling of GFAP and PKC*α* localized Müller cell reactivity to sites of remodeled bipolar cells (Figure 11(f)). In places in which sprouting of bipolar cell dendrites was demonstrated by PKC*α* labelling (Figures 11(d) and 11(f)), we also found prominent Müller cell reactivity spanning the entire retina, with strong GFAP expression in the axial extent of Müller cells (Figure 11(e), white arrows).

## 4. Discussion

This is the first study to report on the attenuation of a secondary wavefront on the descending slope of the scotopic b-wave in rabbits. We show persistent ERG changes and enhanced remodeling of the second- and third-order retinal neurons following retinal detachment and reattachment.

The sole manifestation of functional damage was demonstrated by an attenuation of a peak occurring on the descending slope of the b-wave, which we termed WDS (Figure 1). The WDS parameter was significantly lower in the experimental eyes, compared to the control eyes, while differences in all other a- and b-wave parameters were nonsignificant in both groups. Considering the emphasis we put on the WDS, one may ask why this component was overlooked in previous studies. To the best of our knowledge, a positive deflection riding the descending part of the b-wave was not described and consequently not analyzed in humans. No special recording conditions were needed to produce the WDS. In that sense, our results currently apply only to rabbits. However, the WDS can be seen in other studies that included ERG recordings in rabbits [13]—Figure 1, 2nd row [14, 15]; although, to the best of our knowledge, it has not been previously discussed or explored. One reason might be that the generators of this wavefront are currently unknown, and therefore, at this stage, it is impossible to draw direct structure-function conclusions about this peak. The ideal protocol for recording reproducible WDS is also unclear—some papers do not present representative traces [16] while in others, the WDS is absent [17, 18]. However, in the present study, the WDS was abolished in most of the operated eyes but remained unchanged in the fellow eyes of all of our experimental animals. Therefore, it is not an artefact resulting from anesthesia or the recording protocol but most probably a true biological finding. Positive components of the ERG that span the timing of the WDS are the slow PII component [19], the d.c. component of the b-wave [20], the M-wave [21], and the d- [22] and e-waves [23, 24]. The last two are basically off responses not seen after transient flash and therefore are less likely to produce the WDS. The M-wave is elicited by spot-shaped light stimulation, which is not the case here. Therefore, slow PII component and d.c. wave are possible origins. Because the slow PII is generated by K+ currents in Müller cells, and considering the significant changes in these cells following recovery from RD, these changes might account for the WDS reduction we have observed. Another possible explanation for the WDS appearance would be the latency difference between rod and cone responses, resulting in the early b-wave peak being mostly cone driven, and the later (i.e., the WDS) peak being mostly rod driven. Indeed, the timing and the shape of the isolated cone responses (Figure 4(d)) match the b-wave peak of the mixed rod-cone response (Figure 4(b)), In this case, the WDS decline would be interpreted mainly as attenuation of rod-driven component of the ERG. However, the reason for the manifestation of the WDS in rabbits is undetermined.

We believe this is the first report of its disappearance due to retinal pathology. We found that the WDS to be more sensitive than the classical full-filed ERG (FERG) parameters in rabbits, namely the a- and b-wave amplitudes. Notably, and unlike the recovery of the b-wave amplitude in another study in rabbits [25], a time-dependent trend of recovery was not evidenced for this parameter. The cellular generators of the WDS are unknown, but clearly it is not part of the oscillatory potential complex (Figure 5). It is probably not a pure rod-driven or cone-driven signal as it was absent in both dark-adapted, low-intensity stimulus responses and light-adapted responses, respectively. In order to accurately evaluate the source of the WDS, well-conducted pharmacological trials are warranted to address this issue, for example using N-methyl-D-aspartate to block inner retinal contributions [26–29] or 6-cyano-7-nitroquinoxaline-2,3-dione (CNQX) to block horizontal, some amacrine, and OFF bipolar activity [30].

We studied both functional and structural changes in the rabbit retina, following retinal detachment. No direct comparison to our ERG findings could be found in the literature as previous studies in rabbits were either lacking baseline recordings [16] or utilized in vitro models and were therefore limited to the study of short-term effects [31]. However, Kim et al. [7] found full recovery of the focal ERG b-wave amplitude 7 days postretinal detachment, which is in agreement with our findings. Contrary to their findings, a delay in b-wave implicit time was not found in the present study.

Studies in human patients show typical decreases in a- and b-wave amplitudes after retinal detachment, followed by partial to near complete functional recovery that is evident as early as one week after surgical reattachment [7, 32, 33]. The preserved full-field ERG (FERG) parameters in our study are likely a result of the relatively modest extent of the detached and spontaneous repair of the detachment and tear by the time of the first postoperative recording (Figure 3). Because FERG is a summation of responses from the entire retina, focal functional deficits might have been obscured. Likewise, it is quite possible that had we conducted the first postoperative recording earlier (e.g., 2–3 days after detachment), we would have found significant differences in the FERG responses of the operated eyes.

By day 7 postsurgery, approximately 60% of a detachment has been absorbed, leaving a relative modest extent of a detached retina with much of the retina appearing normal. This is probably why a- and b-wave responses of the scotopic and photopic ERG appeared normal on day 7. Yamamoto et al. [16] showed that there was no significant reduction in ERG a- and b-waves 7 days postsurgery, and Zeng et al. [34] showed that ERG amplitudes were fully recovered by 12 hours postdetachment. Both data support the notion that by day 7 postsurgery, ERG amplitudes (a- and b-waves) should appear normal.

Experiments using pharmacological agents, which block specific cell function, can enable the full evaluation of the cellular sources underlying the functional damage. We theorized that such a drastic abolishment of a sub-b-wave (WDS) would not only be functionally significant (i.e., in ERG) but also anatomically visible. We therefore believed that understanding the cellular changes (morphological, anatomical, etc.) associated with the second- and third-order neurons in detached/reattached retinas might shed a light on the cellular source of the falling phase and serve as a complementary logical approach to guide us to future pharmacological studies. We therefore examined the expression pattern of specific markers of the second- and third-order neurons using immunofluorescence labelling. In agreement with findings previously described by Fisher et al. [5], rod bipolar cells in the experimental retinas were found to be remodeled as their dendrites extended into the ONL which is normally free of PKC-labelled processes. Our findings of extension and sprouting of ON bipolar cells are supported by previously published data [6]; however, the results for the OFF bipolar cells were conflicting. Sakai et al. have shown the retraction of OFF bipolar dendrites from the ONL in a feline retinal detachment model, whereas we found a profound extension of both the dendrites and the axons (Figure 7(f)). These differences can partially be explained by the use of different animal models. Though the feline and rabbit retinas share many morphological similarities, it has been reported that they react differently to detachment [35]. One possible explanation for the different results observed in our study may be attributed to the extent of the secondary degeneration of the neural retina following retinal detachment, which may be different in rabbits [35, 36]. Another possible explanation is the “time-to-reattachment”. In several animal models, the duration of the detachment was relatively short-term, and the retina was reattached within 1–3 days, either naturally or intentionally [5, 34]. In our experimental model, however, the mean time to reattachment was approximately 10–14 days, which may have affected the recovery process of the retina and initiated different cellular responses.

The presynaptic active zone protein Bassoon is essential for photoreceptor ribbon synapse formation in the retina [37, 38]. Therefore, the positive double immunostaining of PKC*α*/Bassoon complex in the extending dendrites in the ONL layer of the experimental retina indicates newly formed connections between the photoreceptors and the bipolar cells. These synapse formation may point toward an increase in the signaling in the outer retina between the photoreceptors and bipolar cell dendrites (Figure 8) [39]. Moreover, since it was found that Bassoon-knockout mice had a persistent reduction in the ERG b-wave [37], our opposite results which illustrate an increase in the Bassoon-immunreactive puncta at the elongated/extended dendrites may therefore suggest the preservation of the overall b-wave at 3 weeks postsurgery.

The plasticity of horizontal cells and to some extent of all amacrine cells has been previously described in retinal diseases in humans and in animal models [9, 11, 40]. Our results, however, did not reveal cellular remodeling of either cell type in the experimental eyes (Figures 9 and 10, resp.). Since reattachment of the retina is known to halt cellular responses [5, 12], it may potentially explain the lack of remodeling observed in our model of retinal attachment-reattachment. Müller cell remodeling has been extensively documented in the literature [5, 11, 12] and is further supported by our results, which demonstrate growth of intermediate filaments from the end feet into the outer retina (Figure 11). Additional analysis of OFF bipolar cells together with cone bipolar cells (yet to be evaluated) should potentially go a long way with pharmacological studies to identify the source of the WDS.

In conclusion, our study shows for the first time the attenuation of a post-b-wave-positive deflection in the falling phase of the b-wave. Cellular remodeling of both the second- and third-order neurons associated with the detachment-reattachment process may potentially contribute to the kinetics of the ERG and shed a light to source of the falling phase. However, the significance of this wavefront should be further explored.

## Figures and Tables

**Table 1:** ERG protocol sequence.

Step	Flash intensity (cd∗s/m^2^)	Number of flashes averaged	Interval between flashes (sec)
1	0.01	10	2
2	3.0	4	10
3	10	4	20
4	Light adaptation: 30 cd/m^2^ for 10 minutes		NA
5	3	32	0.5
6	10	32	0.5
7	3	128	0.032 (flicker)
8	10	128	0.032 (flicker)

## Nomenclature

ERG: Electroretinography
RRD: Rhegmatogenous retinal detachment
IHC: Immunohistochemistry
ONL: Outer nuclear layer.
